# The urinary microbiota composition and functionality of calcium oxalate stone formers

**DOI:** 10.3389/fcimb.2024.1394955

**Published:** 2024-06-07

**Authors:** Jing Xie, Xue-qi Zhang, Ji-nan Guo, Qian Yuan, Ke-feng Xiao, Ye-qing Yuan

**Affiliations:** ^1^ Department of Urology, Shenzhen People’s Hospital (The Second Clinical Medical College, Jinan University, The First Affiliated Hospital, Southern University of Science and Technology), Shenzhen, Guangdong, China; ^2^ Shenzhen Engineering and Technology Center of Minimally Invasive Urology, Shenzhen People’s Hospital, Shenzhen, Guangdong, China

**Keywords:** calcium oxalate stones, functionality, microbiota, shotgun, urine

## Abstract

**Background:**

Accumulated evidences indicate that dysbiosis of the urinary microbiota is associated with kidney stone formation. In the present study, we aimed to investigate the urinary microbiota composition and functionality of patients with calcium oxalate stones and compare it with those of healthy individuals.

**Method:**

We collected bladder urine samples from 68 adult patients with calcium oxalate stones and 54 age-matched healthy controls by transurethral catheterization. 16S rRNA gene and shotgun sequencing were utilized to characterize the urinary microbiota and functionality associated with calcium oxalate stones.

**Results:**

After further exclusion, a total of 100 subjects was finally included and analyzed. The diversity of the urinary microbiota in calcium oxalate stone patients was not significantly different from that of healthy controls. However, the urinary microbiota structure of calcium oxalate stone formers significantly differed from that of healthy controls (PERMANOVA, r = 0.026, P = 0.019). Differential representation of bacteria (e.g., *Bifidobacterium*) and several enriched functional pathways (e.g., threonine biosynthesis) were identified in the urine of calcium oxalate stone patients.

**Conclusion:**

Our results showed significantly different urinary microbiota structure and several enriched functional pathways in calcium oxalate stone patients, which provide new insight into the pathogenesis of calcium oxalate stones.

## Introduction

Nephrolithiasis is a common disease of urinary tract, with a global rising prevalence and a recurrence rate of 50% within the first 5 years of the initial episode (Wang et al., 2022). Despite the great advances in the surgical treatment of urolithiasis, the mechanism of Calcium oxalate (CaOx) stone formation and development remains elusive, and medical prophylaxis and therapies have not improved substantially during the past decades (Rodgers and Trinchieri, 2023).

For a long time, urinary supersaturation and crystallization with calcium and oxalate was considered the main mechanism of stone formation. However, recent studies showed that there is no significant difference in urine chemistry of recurrent stone formers and healthy controls, indicating that urinary supersaturation alone is not sufficient to explain the formation of CaOx stones (Wang et al., 2021).

With the rapid development of high-throughput sequencing, the urinary tract has been proved to possess a diverse population of microbes, which is critical for the maintenance of urogenital homeostasis. Imbalances in the urinary microbiome can contribute to several urologic diseases, such as urinary tract infection, incontinence, and genitourinary cancer (Colella et al., 2023). In recent years, accumulated evidences have shown dysbiosis of urinary microbes was associated with the formation of kidney stones (Miller et al., 2022). In our preliminary study, we observed a marked dysbiosis of urinary microbiota in male patients with calcium-based kidney stones (Xie et al., 2020). However, the study recruited patients of mixed stone types with a small sample size, and female subjects were not included. In addition, we only utilized 16S rRNA gene sequencing to predict the functional pathways, which was not sufficient to explore the functionality of the urinary microbiome.

In the present study, we focused on CaOx stones, recruited both male and female individuals, and utilized 16S rRNA gene and shotgun sequencing to characterize the urinary microbiota and functionality associated with CaOx stones. The objective of our research was to (1) identify the key microbes that significantly differ between CaOx stone formers and healthy individuals; (2) explore functional pathways that significantly enriched in the urinary microbiome of CaOx stone formers.

## Materials and methods

### Participant recruitment

The study protocol was approved by Ethical Review Board of Shenzhen People’s Hospital, Second Clinical Medical College, Jinan University (LL-KY-2020337). Informed consents were obtained from all subjects. Between April 2021 and July 2022, a total of 122 adults were recruited at our hospital, including 68 kidney stone formers and 54 age-matched healthy volunteers. Kidney stones were initially evaluated by ultrasonography, abdominal plain film, intravenous pyelography and computed tomography, and confirmed during endoscopic surgery. We initially screened the CaOx stone formers by X-ray findings and value measurement on CT. The chemical composition of surgically removed stones was further confirmed by infrared spectroscopy analysis. Both calcium oxalate monohydrate and calcium oxalate dihydrate were considered as calcium oxalate composition, while other mineralogies such as carbonate apatite and uric acid were not considered.

For kidney stone formers, exclusion criteria included stones of calcium oxalate composition <90%, concurrent ureteral calculus, and moderate to severe hydronephrosis. For healthy participants, exclusion criteria included personal history of urinary stones, episodes of renal colic or imaging confirmed urinary stones. All healthy controls underwent ultrasonography or computed tomography to exclude asymptomatic renal calculus. Excluded from both groups were subjects with urinary tract infections or positive urine culture, antibiotic use within 30 days, indwelling catheter or ureteral stent, congenital abnormalities of the urinary tract, history of major urological surgery, malignant tumors, diabetes, chronic kidney disease, autoimmune disease and age (< 20 years or >70 years old).

### Sample collection and processing

Bladder urine samples were collected by transurethral catheterization from all participants in a fasting state. All samples were obtained prior to antibiotic use, and the volume of each urine sample was approximately 50 ml. To remove host cell fragment, the urine samples were immediately centrifuged 300g for 5 minutes at 4°C. Then the supernatant was collected and centrifuged 12,000g for 15 minutes at 4°C. Pellets were re-suspended, mixed with DNA-free phosphate buffered saline, and stored in sterile containers at -80°C within 2 h from collection. The supernatant collection and pellets resuspension were performed on a clean workbench, following strict aseptic procedures.

### 16S rRNA microbial profiling analyses

Genomic DNA was extracted from urine samples using MagPure Stool DNA KF Kit B (MAGEN, China). The DNA concentration was quantified by Qubit^®^ 2.0 Fluorometer (Life Technologies, USA), and DNA integrity was verified with agarose gel electrophoresis. The DNA extractions were stored at −20°C until further processing. Polymerase chain reaction was performed to amplify the V3-V4 region of the 16S rRNA gene, with primers shown as follows: V3-V4-338F: 5′- ACTCCTACGGGAGGCAGCAG- 3′ and 806R: 5′- GGACTACHVGGGTWTCTAAT -3′. The amplicons were purified using the AMPure beads (Axygen, USA), and barcoded libraries were sequenced on Illumina Hiseq2500 platform.

From the raw sequencing data, low-quality reads were eliminated and clean reads were merged to tags using FLASH software (v1.2.11) (He et al., 2013). Then the sequences with 100% similarity were assigned to the same operational taxonomic units (OTUs) using Divisive Amplicon Denoising Algorithm 2 in software QIIME2, a newer algorithm compared to traditional Usearch with 97% similarity. RDP Classifier (v2.2) (Wang et al., 2007) was used to taxonomically classify the representative sequences of each OTU, based on the Greengenes database (V201305). For α−diversity, observed species, ACE index, chao 1 index, Simpson index, Shannon index and Good-coverage index were calculated by Mothur (v.1.31.2) (Schloss et al., 2009). For β−diversity, both weighted and unweighted UniFrac distances were conducted using QIIME (v1.80), and shown by the principal coordinate analysis (PCoA) (Caporaso et al., 2010). LEfSe analysis (Segata et al., 2011) was performed to find biomarkers differentially represented between the KS and HC groups.

### Shotgun metagenomics analysis of urine samples

DNA extracted from 30 urine samples selected to include 15 KS subjects and 15 controls, representative of the average composition of KS individuals and controls, was employed to perform shotgun metagenomics sequencing. The DNA extractions were fragmented to 550–650 bp by a Focused-ultrasonicator (Covaris, USA). The sample preparation was performed using BGI Optimal DNA Library Prep Kit (BGI, China), and barcoded libraries were sequenced on a MGI MGISEQ-2000 platform. All raw data were pre-processed by SOAPnuke v.2.2.1 (Chen et al., 2018), and the trimmed reads were mapped to the host genome using SOAP2 software to identify and remove host originated reads. High-quality reads were *de novo* assembled using MEGAHIT software (Li et al., 2015), and assembled contigs with length less than 300 bp were discarded. Genes were predicted over contigs by using MetaGeneMark (Zhu et al., 2010). Redundant genes were removed using CD-HIT (Fu et al., 2012) with the identity and coverage cutoff 95%, 90%, respectively. To generate the annotation information, the protein sequences of genes were aligned against the functional database (BacMet, CARD, CAZy, COG, KEGG, EggNOG and Swiss-Prot), using DIAMOND (Buchfink et al., 2015) with an E value cutoff of 1e−5. We identified differentially enriched KEGG pathways according to reporter scores (Patil and Nielsen, 2005), and the detection threshold for significance was an absolute value of reporter score of 1.65 or higher.

### Statistical analyses

Statistical analyses were performed using the SPSS (version 21.0) and R software (version 4.1.3), and p values < 0.05 was considered as statistically significant. Clinical continuous variables were compared using a student’s t test, while categorical variables were analyzed via Pearson’s chi-square test or Fisher’s Exact Test. The continuous variables were expressed as mean ± standard deviation. For 16S rRNA sequencing, α-diversity analysis were performed with mothur (v.1.31.2) using Wilcoxon rank-sum test, while β−diversity was calculated by software QIIME (v1.80). For shotgun metagenomics, both α-diversity and β−diversity was calculated by python package of R project, using scikit-bio and scipy, respectively.

## Results

### General characteristics of CaOx stone patients and controls

Urine samples were collected from a total of 122 subjects. In the CaOx stone group, nine subjects were further excluded for DNA library construction failure, ten for CaOx composition <90% and one for not performing infrared spectroscopy. In the control group, two subjects were excluded for DNA library construction failure. The demographic and clinical data of the rest 100 subjects was listed in **Table 1**. There was no significant difference between CaOx stone formers and healthy controls in terms of age, gender, and body mass index. Although comorbidities such as hypertension, hyperuricemia and hyperlipidemia were more common in CaOx stone patients, they did not reach statistical significance. The majority of stone formers were first onset (40/48, 83.3%) and eight patients were recurrent. In the stone formers group, 60.4% (29/48) was pure CaOx and the rest was mixed with carbonate apatite or anhydrous uric acid (<10%).

**Table 1:** Demographic and clinical data for the calcium oxalate stone formers and healthy subjects.

	Stone formers (n=48)	Healthy controls (n=52)	P values
Age, years	45.1±12.3	41.3±13.6	0.143
Female	8	10	0.739
BMI, kg/m^2^	24.8±2.92	23.6±3.16	0.055
Prior USD	8	0	NA
Comorbidities			
Hypertension	7	4	0.435
Diabetes	0	0	1.0
Hyperuricemia	11	8	0.337
Hyperlipidemia	5	6	0.858
Stone composition			NA
COM	3	NA	
COD	6	NA	
COM+COD	39	NA	
Urine pH	6.1±0.71	6.2±0.71	0.442

BMI, Body mass index; USD, Urinary stone disease; COM, Calcium oxalate morlohydrate; COD, Calcium oxalate dihydrate.

### Biodiversity of the urinary microbiota

**Figure 1:**
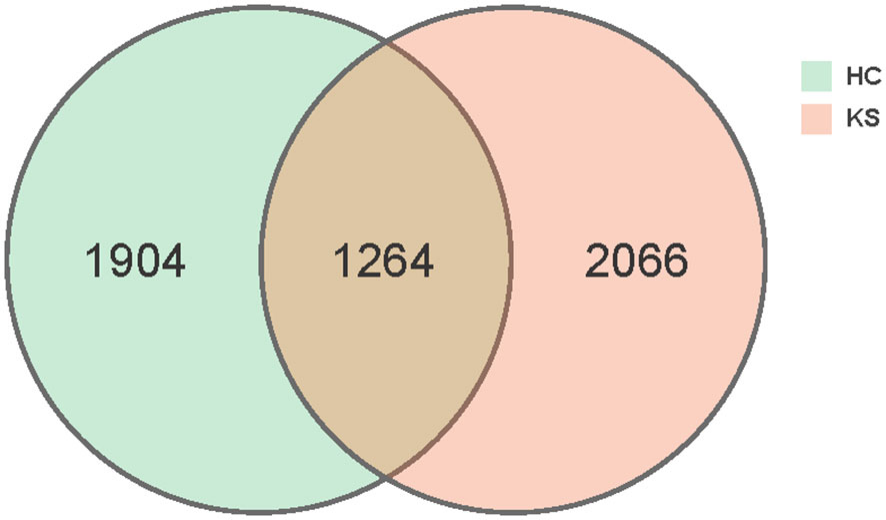
Venn diagram. A total of 5234 OTUs were detected with 2066 OTUs in KS samples only, 1904 OTUs in HC samples only and 1264 OTUs overlapping.

In total, we obtained 15,829,456 clean reads from the 100 urine samples. The median number of reads in CaOx stone patients was 157,824 and in healthy controls was 158,729. The reads were classified into 5234 unique OTUs at 100% similarity level that were used for further analysis. We defined two groups according to the kidney stone status: KS represents patients with CaOx stones, while HC represents healthy volunteers. There was substantial overlap in the OTUs composition between KS and HC groups (**Figure 1**). The average OTUs showed no significant difference between KS and HC groups, with an average of 177 OTUs in KS group and 170 OTUs in HC group, respectively (P =0.625).

**Figure 2:**
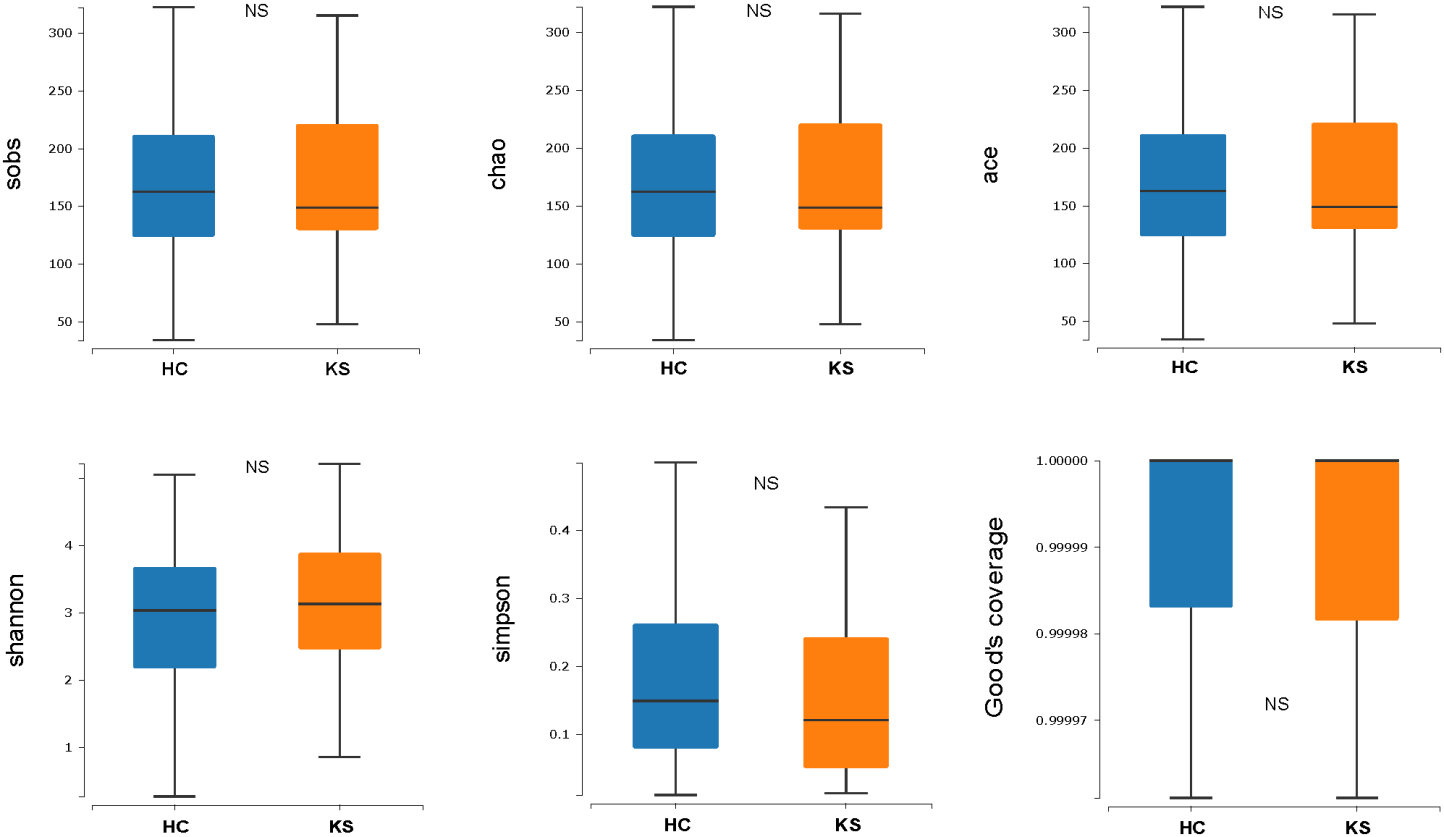
Microbial α−diversity of urine samples. The α−diversity indices include sobs index, chao index, ACE index, Shannon diversity index and Simpson diversity index and Good’s coverage index. All index showed no significant difference between KS and HC group.

**Figure 3:**
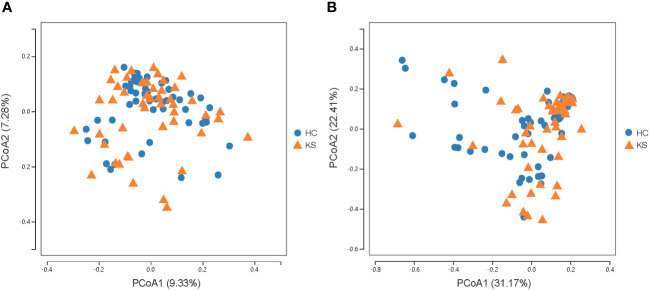
Microbial β−diversity analysis. PCoA plot of unweighted **(A)** and weighted **(B)** UniFrac metrics for KS (orange triangles) and HC (blue dots) groups. The sample scatters of KS and HC groups are not apparently separated.

For α−diversity, the values of Good’s coverage index of all libraries were > 99%. The α−diversity indices, including sobs index, chao index, ACE index, Shannon diversity index and Simpson diversity index, were not significantly different between KS and HC groups (**Figure 2**). For β−diversity, we applied unweighted and weighted principal coordinate analysis (PCoA) to display discrepancy between the two groups. Evident clusterization in each group was observed, although the sample scatters of KS and HC groups are not apparently separated (**Figure 3**). PERMANOVA was also performed, and we found the urinary microbiota structure of KS patients and controls was significantly different (unweighted unifrac, r = 0.019, P = 0.002; weighted unifrac, r = 0.026, P = 0.019, respectively).

### Taxonomic analysis of urinary microbiota

**Figure 4:**
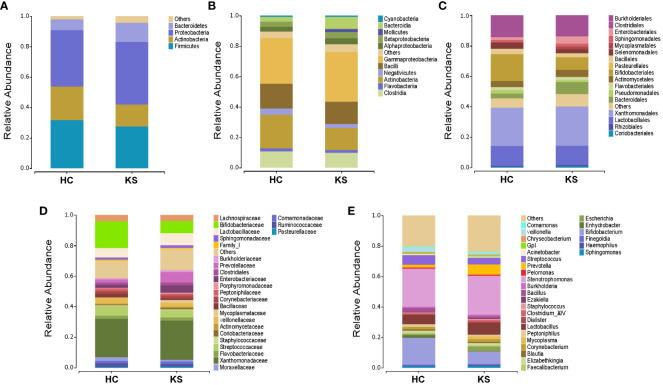
Bacterial average relative abundance in KS and HC groups at different taxonomic levels. **(A)** phylum, **(B)** class, **(C)** order, **(D)** family, **(E)** genus. Average distribution of taxa was represented by bar graphs. Unclassified genera or genera with a relative abundance <1% were grouped as “Other”.

To identify the differentially represented taxa in CaOx stone patients and healthy controls, we compared the relative abundance of microbiota between the two groups at different taxonomic levels (**Figure 4**). At phylum level, it showed statistically significant difference between KS and HC groups in the average representation of Bacteroidetes, Fusobacteria and Chloroflexi. KS group showed a higher average abundance of Bacteroidetes (12.5% *vs* 7.2%, p<0.01) and Chloroflexi (0.1% *vs* 0.03%, P<0.01), and a lower average abundance of Fusobacteria (0.1% *vs* 0.6%, p<0.01). Significant abundance differences of numerous taxa were also noted between KS and HC groups at other taxonomic levels (**Supplementary Table 1**). At genus level, the average abundance of *Bifidobacterium* was significantly higher in HC group compared to KS group (17.7% *vs* 8.0%, p=0.03).

### Specific urinary genera associated with CaOx stones

**Figure 5:**
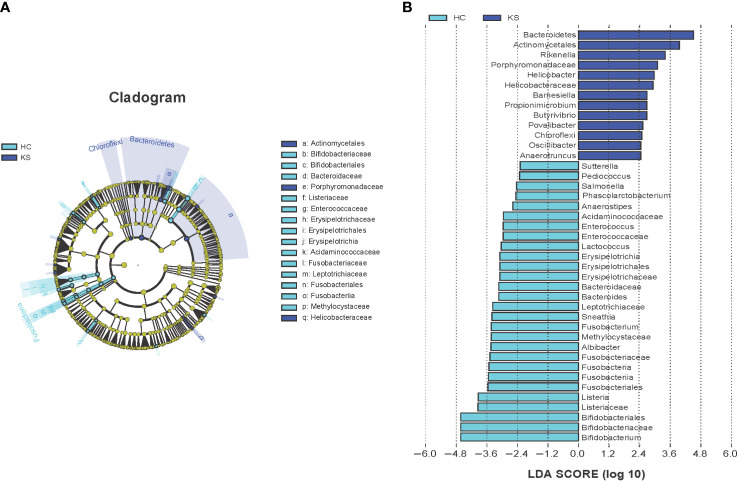
Cladogram **(A)** and LEfSe analyses **(B)** of microbiomes between KS (blue) and HC (cyan) groups. Taxa in graph were with LDA score threshold >2.0 and statistically significant (p <0.05).

We further applied LEfSe analysis to confirm the differentially represented taxa in the urine of CaOx stone patients and controls. Taxa were considered differentially represented with P < 0.05 in Wilcoxon test and logarithmic LDA score more than 2.0. In total, LEfSe identified 41 discriminative features with significant different relative abundance between KS and HC groups (**Figure 5**). The taxa at genus level that differentiated the two groups most were *Rikenella* in KS group and *Bifidobacterium* in HC group. We further used LEfSe to analyze the male subgroups, and found that *Bifidobacterium* remained the most significantly different genera in male healthy controls (**Supplementary Figure 1**).

### Functional analysis of urinary microbiota through shotgun metagenomics

**Figure 6:**
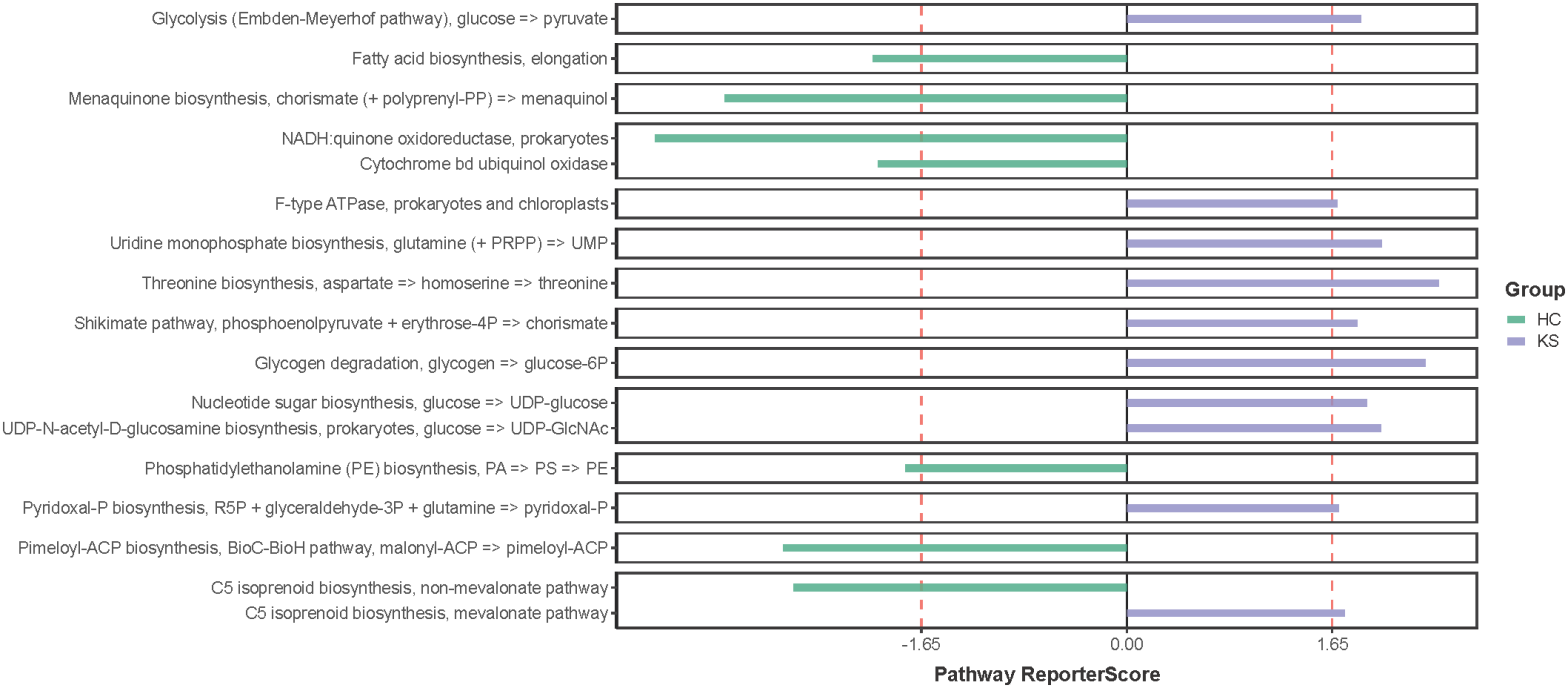
KEGG pathway enrichment analysis. Several differentially represented functional molecules were identified between KS and HC groups.

Having observed different bacterial community in the urine of CaOx stone patients, we further applied shotgun metagenomics sequencing to evaluate whether the different urinary microbiota was associated with specific functionality alterations. After removal of the host originated reads, a total number of 14,783,970 filtered reads (range 221,102~1,934,456) was obtained. The functional pathways of urinary microbiota in KS and HC samples were inferred using KEGG pathway enrichment analysis. Compared to HC group, the significantly enriched KEGG molecules in KS groups included threonine biosynthesis, glycogen degradation and uridine monophosphate biosynthesis (**Figure 6**).

## Discussion

In the present study, we utilized 16S rRNA gene and shotgun metagenomics sequencing to explore the urinary microbiota and functionality in CaOx stone formers. Interestingly, our results showed that the urinary species diversity of CaOx stone formers was not significantly different from healthy controls. However, the urinary microbiota structure of CaOx stone patients and healthy individuals was distinctly different. In addition, several differentially represented taxa and functional molecules were identified between KS and HC groups.

Pelvic urine is an ideal body fluid to study the mechanisms of kidney stone formation. However, due to its invasive and cumbersome process, it is not feasible to collect pelvic urine in clinical practice or largescale researches. In 2020, Dornbier reported that there was no significant difference in the microbial composition of bladder urine and upper tract urine in urinary stone patients (Dornbier et al., 2020). It is worth noting that several confounding factors were not excluded in that study, such as antibiotic use and ureteral stent placement, which may affect the credibility of the conclusions.

In our previous research, we set strict inclusion criteria and our results showed the overall bacterial composition and predicted functional pathways of bladder urine was similar to that of renal pelvis urine in kidney stone patients (Xie et al., 2020). In a subsequent study, the urinary microbiome in the kidney pelvis was also proved to be similar to that in the bladder urine after strict disinfection of the bladder (Liu et al., 2020). Moreover, Lemberger compared the bacteria composition between catheterized urine and kidney stones using 16S rRNA gene amplicon sequencing, and found that the microbiome of stones was well reflected in the corresponding patients’ urine sample (Lemberger et al., 2023). Thus, these studies suggested that catheterized bladder urine was a viable alternative to pelvic urine in microbiome research.

Consistent with previous studies, we observed that the urinary microbiota structure of CaOx formers was significantly different from that of healthy individuals. At genus level, the most differentially represented taxa were *Rikenella* in KS group, and *Bifidobacterium* in healthy controls. *Rikenella*, belonging to the phylum Bacteroidota, has been shown to be associated with inflammatory and involved in the formation of short‐chain fatty acids (Cai et al., 2023). Short‐chain fatty acids was reported to alleviate CaOx crystal formation and adhesion to kidney cells through immunomodulation (Jin et al., 2021). *Bifidobacterium* are a group of Gram-positive, nonspore-forming, strictly anaerobic bacteria, which are widely existed in the intestinal tract, feces, oral cavity, and breast milk. *Bifidobacterium* synthesize various vitamins, produce many sorts of short-chain fatty acids such as acetic acid and propionic acid, and are now widely used as probiotics to promote human health (Vera-Santander et al., 2023). It has been shown that black South Africans have a very low prevalence of kidney stones (<1%) and that this population has a low abundance of *Oxalobacter formigenes* in the gut, whereas the abundance of *Bifidobacterium* and *Lactobacilli* is significantly increased. Thus, it was speculated that *Bifidobacterium* and *Lactobacilli* have a more protective role against stone formation compared to *Oxalobacter formigenes (* Magwira et al, 2012). Federici et al. found that several *Bifidobacterium* strains exhibited different degrees of oxalate-degrading activity *in vitro* experiments, the strongest of which was *B. lactis DSM 10140* (degrading 60.6% of available oxalate) (Federici et al., 2004). Moreover, a recent study has shown that *Bifidobacterium* was more abundant in the gut of no stone individuals than that of incidental stone patients (Kim et al., 2022). Interestingly, it has been reported that the genera *Bifidobacterium* were more predominant in females, which may contribute to the lower incidence of urinary stones in women (Nickel et al., 2022). However, in a recently conducted randomized clinical trial, urinary oxalic acid levels were not significantly reduced in 54 patients with kidney stones after 4 weeks of administration of a probiotic containing *Bifidobacterium animalis*, and the authors thought it might be related to failure of intestinal colonization of the probiotic (Tavasoli et al., 2021). In the present study, we observed that the average abundance of *Bifidobacterium* was significantly higher in HC group compared to KS group. Together with the above research, our results suggested that *Bifidobacterium* is closely associated with the formation of CaOx stones, and the potential mechanisms need to be further elucidated.

We also tried to explore functional pathways that significantly enriched in the urinary microbiome of CaOx stone formers by utilizing shotgun sequencing. Our results showed that threonine biosynthesis was significantly enriched in the urine of CaOx stone formers. Interestingly, a previous study has showed that glycine, serine and threonine metabolism was closely associated with kidney stone formation, which was in accordance with our findings (Duan et al., 2020). Several studies have shown a close relationship between abnormal levels of amino acids in human urine and the formation of urinary stones. In patients with cystinuria, there is a marked increase in urinary excretion of low-solubility cystine, which in turn leads to the formation of specific cystine stones (D’Ambrosio et al., 2022). Early *in vitro* experiments have shown that glutamate significantly promotes calcium oxalate crystallization in the urine, whereas aspartate and alanine have less effect on calcium oxalate crystallization (Azoury et al., 1986). In a recent study, hydroxyproline was shown to promote calcium oxalate stone formation in rat kidney by mediating inflammatory and macrophage immune-related signaling pathways (Kumar et al., 2022). On the other hand, glycine was shown to inhibit calcium oxalate stone formation by decreasing urinary oxalate excretion and increasing citrate secretion (Lan et al., 2021). Our study suggests that threonine synthesis is closely associated with CaOx stone formation, although the exact mechanisms require further investigation

The present study had several limitations. First, although we focused on calcium oxalate stones patients with relatively adequate sample sizes, all participants were Chinese and limited generalizability. Second, we tried to conduct a comprehensive study by recruiting both females and males in line with our inclusion criteria, but the sample size of female participants was still inadequate, mainly due to the higher positive rate of urine routine tests. Third, since the vast majority of CaOx patients were first onset and 24 h urine analyses were not performed, we were unable to evaluate the association of urine microbiota and urine lithogenesis factors. Finally, our study only revealed a close association between the urinary microbiome and CaOx stones, the exact role of urinary microbiota and related mechanisms need to be elucidated.

## Conclusions

In conclusion, our results showed significantly different urinary microbiota structure in CaOx stone patients compared to healthy individuals, and revealed several functional pathways potentially associated with formation of CaOx stones. Our findings provide new insight into the pathogenesis of CaOx stones and potential targets for the intervention of the disease.

## Data availability statement

The original contributions presented in the study are included in the article/**Supplementary Material**. Further inquiries can be directed to the corresponding author.

## Ethics statement

The study was approved by Ethical Review Board of Shenzhen People's Hospital, Second Clinical Medical College, Jinan University (LL-KY-2020337). Informed consent was obtained from all participants.

## Author contributions

JX: Writing – original draft, Project administration, Funding acquisition, Conceptualization. X-QZ: Writing – review & editing, Investigation, Formal analysis, Data curation, Conceptualization. J-NG: Writing – original draft, Methodology, Formal analysis, Data curation, Conceptualization. QY: Writing – review & editing, Methodology, Investigation, Conceptualization. K-FX: Writing – review & editing, Supervision, Conceptualization. Y-2QY: Writing – review & editing, Validation, Supervision, Project administration, Conceptualization.

## Supplementary material

Supplementary Figure 1 Bifidobacterium remained the most significantly different genera in male healthy controls.
