# Supplementary figures and images for: The urinary microbiota composition and functionality of calcium oxalate stone formers
Source: Front Cell Infect Microbiol. 2024 Jun 7;14:1394955. doi: 10.3389/fcimb.2024.1394955 (PMC11190077; doi:10.3389/fcimb.2024.1394955)

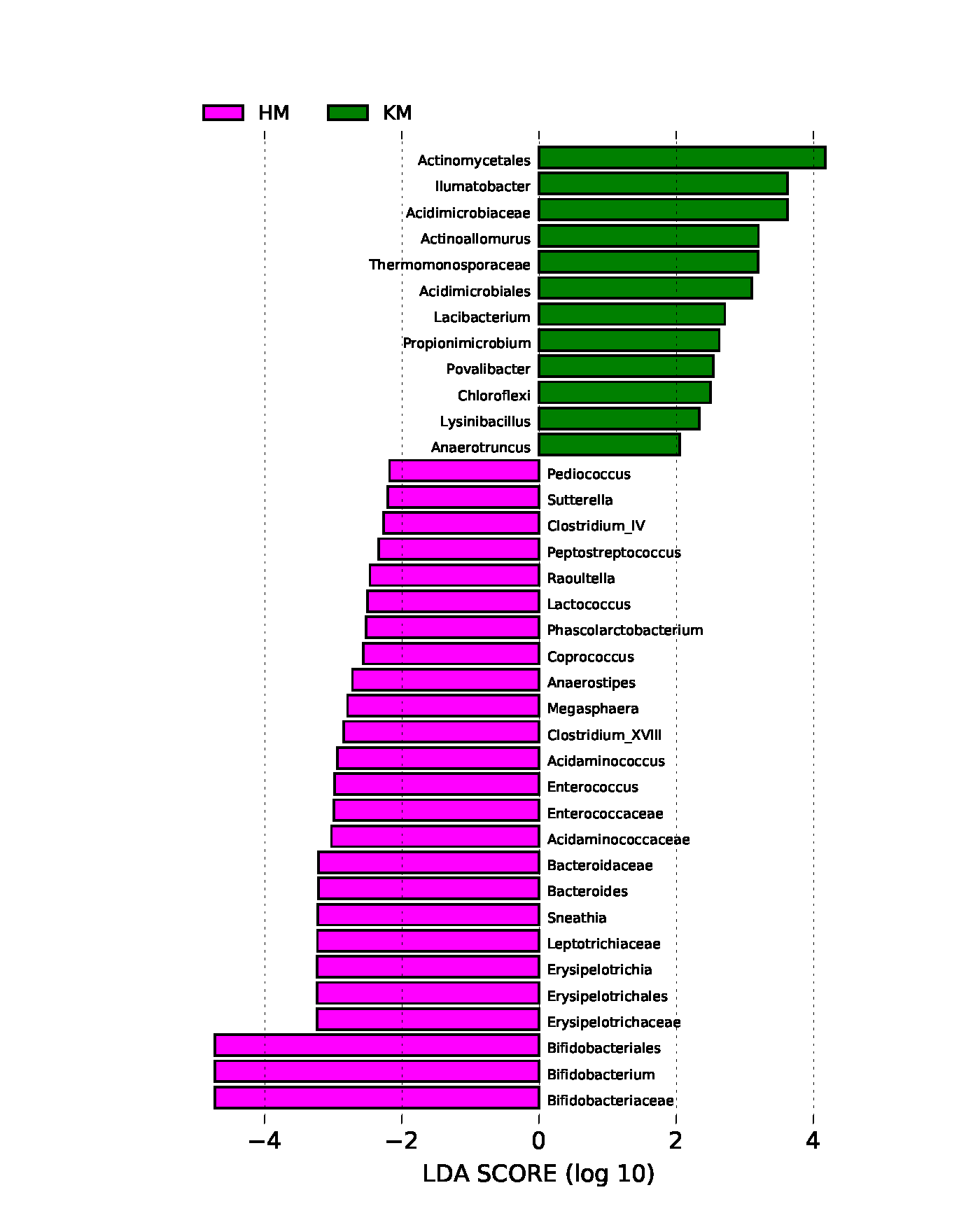

Supplement: Supplementary Figure 1 — Bifidobacterium remained the most significantly different genera in male healthy controls. [file Image_1.tif]
