# Supplementary material for: The urinary microbiota composition and functionality of calcium oxalate stone formers
Source: Front Cell Infect Microbiol. 2024 Jun 7;14:1394955. doi: 10.3389/fcimb.2024.1394955 (PMC11190077; doi:10.3389/fcimb.2024.1394955)
Supplement: Supplementary file 2 [file DataSheet_1.docx]

**Supplementary Table 1 Comparison of average relative abundance of bladder urine microbiome in kidney stone patients and healthy subjects at different taxonomic levels.**

| **Taxa** | | **Average abundance (%)** | | | **Prevalence (%)** | |
| --- | --- | --- | --- | --- | --- | --- |
|  |  | **P value** | **HC** | **KS** | **HC** | **KS** |
| Phylum | Bacteroidetes | 0.003 | 7.179 | 12.518 | 100 | 100 |
|  | Fusobacteria | 0.005 | 0.590 | 0.123 | 58 | 29 |
|  | Chloroflexi | 0.008 | 0.031 | 0.103 | 15 | 42 |
| Family | Porphyromonadaceae | 0.004 | 0.526 | 0.858 | 96 | 96 |
|  | Leptotrichiaceae | 0.006 | 0.232 | 0.001 | 19 | 2 |
|  | Enterococcaceae | 0.008 | 0.208 | 0.151 | 63 | 35 |
|  | Acidaminococcaceae | 0.010 | 0.229 | 0.107 | 62 | 40 |
|  | Bacteroidaceae | 0.015 | 0.602 | 0.406 | 94 | 83 |
|  | Fusobacteriaceae | 0.017 | 0.358 | 0.123 | 52 | 27 |
|  | Bifidobacteriaceae | 0.028 | 17.726 | 7.958 | 100 | 100 |
|  | Erysipelotrichaceae | 0.028 | 0.451 | 0.230 | 88 | 73 |
|  | Listeriaceae | 0.029 | 0.006 | 0.000 | 10 | 0 |
|  | Iamiaceae | 0.031 | 0.003 | 0.130 | 4 | 17 |
|  | Methylocystaceae | 0.035 | 0.040 | 0.001 | 13 | 2 |
|  | Nocardioidaceae | 0.038 | 0.014 | 0.081 | 19 | 35 |
|  | Helicobacteraceae | 0.044 | 0.003 | 0.01 | 13 | 29 |
| Genus | Phascolarctobacterium | 0.001 | 0.095 | 0.056 | 56 | 23 |
|  | Sutterella | 0.004 | 0.038 | 0.014 | 40 | 15 |
|  | Anaerotruncus | 0.006 | 0.001 | 0.007 | 10 | 29 |
|  | Propionimicrobium | 0.009 | 0.084 | 0.106 | 15 | 38 |
|  | Pediococcus | 0.009 | 0.021 | 0.000 | 13 | 0 |
|  | Bacteroides | 0.015 | 0.602 | 0.406 | 94 | 83 |
|  | Fusobacterium | 0.016 | 0.358 | 0.122 | 52 | 27 |
|  | Povalibacter | 0.018 | 0.000 | 0.017 | 0 | 10 |
|  | Sneathia | 0.021 | 0.229 | 0.001 | 15 | 2 |
|  | Enterococcus | 0.026 | 0.202 | 0.150 | 58 | 35 |
|  | Bifidobacterium | 0.028 | 17.726 | 7.955 | 100 | 100 |
|  | Lactococcus | 0.029 | 0.001 | 0.000 | 10 | 0 |
|  | Listeria | 0.029 | 0.006 | 0.000 | 10 | 0 |
|  | Albibacter | 0.035 | 0.040 | 0.001 | 13 | 2 |
|  | Butyrivibrio | 0.035 | 0.000 | 0.005 | 0 | 8 |
|  | Rikenella | 0.035 | 0.000 | 0.002 | 0 | 8 |
|  | Barnesiella | 0.039 | 0.206 | 0.273 | 90 | 92 |
|  | Oscillibacter | 0.042 | 0.036 | 0.068 | 48 | 58 |
|  | Anaerostipes | 0.043 | 0.120 | 0.050 | 52 | 33 |
|  | Helicobacter | 0.044 | 0.001 | 0.007 | 8 | 21 |
| Species | Prevotella_timonensis | <0.001 | 0.096 | 2.770 | 23 | 58 |
|  | Streptococcus_danieliae | <0.001 | 0.327 | 0.084 | 38 | 6 |
|  | Phascolarctobacterium_faecium | 0.002 | 0.063 | 0.045 | 48 | 19 |
|  | Sutterella_stercoricanis | 0.004 | 0.038 | 0.014 | 40 | 15 |
|  | Pseudomonas_aeruginosa | 0.004 | 0.024 | 0.009 | 40 | 15 |
|  | Anaerotruncus_colihominis | 0.006 | 0.001 | 0.007 | 10 | 29 |
|  | Paracoccus_sphaerophysae | 0.007 | 0.045 | 0.006 | 29 | 8 |
|  | Actinomyces_radingae | 0.008 | 0.005 | 0.061 | 6 | 25 |
|  | Propionimicrobium_lymphophilum | 0.009 | 0.084 | 0.106 | 15 | 38 |
|  | Enterococcus_mundtii | 0.009 | 0.039 | 0.000 | 13 | 0 |
|  | Nocardioides_islandensis | 0.009 | 0.000 | 0.004 | 0 | 13 |
|  | Bifidobacterium_animalis | 0.011 | 0.222 | 0.143 | 65 | 35 |
|  | Bacteroides_dorei | 0.012 | 0.088 | 0.042 | 71 | 50 |
|  | Lactobacillus_fermentum | 0.014 | 0.006 | 0.004 | 17 | 2 |
|  | Parabacteroides_merdae | 0.015 | 0.031 | 0.004 | 31 | 13 |
|  | Coprococcus_comes | 0.016 | 0.078 | 0.060 | 35 | 13 |
|  | Acinetobacter_lwoffii | 0.016 | 0.022 | 0.000 | 12 | 0 |
|  | Paraprevotella_clara | 0.017 | 0.035 | 0.009 | 31 | 13 |
|  | Megasphaera_elsdenii | 0.017 | 0.094 | 0.021 | 27 | 8 |
|  | Povalibacter_uvarum | 0.018 | 0.000 | 0.017 | 0 | 10 |
|  | Sneathia_sanguinegens | 0.021 | 0.229 | 0.001 | 15 | 2 |
|  | Ruminococcus_callidus | 0.021 | 0.051 | 0.007 | 19 | 4 |
|  | Oscillibacter_valericigenes | 0.025 | 0.020 | 0.028 | 17 | 35 |
|  | Listeria_ivanovii | 0.029 | 0.006 | 0.000 | 10 | 0 |
|  | Bacteroides_fragilis | 0.030 | 0.072 | 0.022 | 31 | 13 |
|  | Albibacter_methylovorans | 0.035 | 0.040 | 0.001 | 13 | 2 |
|  | Rikenella_microfusus | 0.035 | 0.000 | 0.002 | 0 | 8 |
|  | Prevotella_stercorea | 0.039 | 0.080 | 0.068 | 52 | 29 |
|  | Blautia_schinkii | 0.042 | 0.036 | 0.008 | 13 | 2 |
|  | Clostridium_scindens | 0.042 | 0.016 | 0.034 | 17 | 33 |
|  | Anaerostipes_hadrus | 0.044 | 0.117 | 0.046 | 52 | 33 |
